# Bioinformatics-based identification of potential genes in the thalamus associated with chronic orofacial and somatic inflammatory pain

**DOI:** 10.3389/fnins.2026.1879002

**Published:** 2026-08-14

**Authors:** Yan Cheng, Juan-Juan Yuan, Jing Liu, Xiao-Yu Guo, Yuan-Ming Li, Di-Yin Zhang, Yu Zhang, Li-Tian Yin

**Affiliations:** 1 Key Laboratory of Cellular Physiology, Ministry of Education, Shanxi Key Laboratory of Aging-related Diseases Mechanisms and Translation, Department of Physiology, Shanxi Medical University, Taiyuan, China; 2 Department of Endocrinology, The Second Hospital of Shanxi Medical University, Taiyuan, China; 3 Biomedical and Health Laboratory in Shanxi Province, Shanxi University, Taiyuan, China

**Keywords:** bioinformatic analysis, hub genes, immune regulation, inflammatory pain, thalamus

## Abstract

**Background:**

The thalamus is one of the structures that receives projections from multiple ascending pain pathways. Although the transmission of orofacial and somatic pain involves different nuclear clusters and nerve pathways, the thalamic neuroimmune alterations across these different pain modalities remain poorly understood. This work aimed to investigate alterations in the neuroimmune mechanisms of the thalamus under chronic orofacial and somatic inflammatory pain conditions via bioinformatics analysis and to identify novel therapeutic targets for chronic pain management.

**Methods:**

To illuminate the potential molecular mechanisms in inflammatory pain, we compared thalamic mRNA expression levels between Complete Freund’s Adjuvant (CFA) induced chronic orofacial pain and somatic inflammatory pain in rats. Bioinformatic analyses were conducted to determine functional pathway enrichment and protein–protein network analyses.

**Results:**

The overlapping of differentially expressed genes (DEGs) between chronic orofacial pain and plantar pain included 13 genes. Gene Ontology (GO) analysis revealed that the DEGs were mainly related to inflammatory response, immune system process, and cytokine activity. The Kyoto Encyclopedia of Genes and Genomes (KEGG) pathway analysis highlighted a prominent enrichment in the IL-17 signaling pathway. The RT-qPCR was used to identify the mRNA expressions of DEGs in the thalamus of rats with chronic orofacial and plantar inflammatory pain, thereby validating our analytical results.

**Conclusion:**

Our study identified candidate hub genes that are associated with the pathogenesis of chronic orofacial and somatic inflammatory pain in the thalamus, which provides preliminary insights into potential alterations in immune mechanisms within the thalamus under different pain conditions.

## 1 Introduction

Inflammatory pain is the body’s defensive response to harmful stimuli. However, excessive or persistent inflammatory pain not only increases the psychological burden on patients, but may also transition to chronic pain and impair their quality of life (Liang et al., 2020). Although the mechanisms of pain remain unclear, most studies concentrate on pain conduction pathways, including the dorsal root ganglion and spinal dorsal horn, as well as pain-related emotions, whereas the role of the thalamus has received little attention (Ou et al., 2024). The thalamus acts as a crucial relay station in the central nervous system, responsible for transmitting signals from diverse body regions to the cerebral cortex. It is instrumental in conveying nociceptive information to brain areas associated with pain perception, making it a vital component of the ascending pain modulatory system (Bonin et al., 2023). With advancements in research technologies, the core role of the thalamus in pain processing has been extensively re-evaluated. The thalamus is not only an obligatory relay station for ascending sensory inputs but also a key integrative center that integrates pain signals across both orofacial and somatic regions, thereby shaping the multidimensional experience of pain (Galdino et al., 2024; Robertson et al., 2024).

Mechanical hyperalgesia of the orofacial area can be induced by oral and facial inflammation through activation of primary and secondary sensory neurons in the trigeminal nerve (Rotpenpian and Yakkaphan, 2021). Chronic pain not only causes physical suffering but is also frequently accompanied by emotional disorders, such as anxiety and depression. Recent research has revealed that the paraventricular nucleus of the thalamus (PVT) may play a crucial role as an integrative center in this process, highlighting the functional heterogeneity of PVT in regulating chronic pain and emotion-related behaviors. Consequently, the thalamus is redefined not only as a passive sensory relay, but also as a central hub actively participating in the integration of pain and emotional states (Li et al., 2025). Studies have found that in the model of inflammatory pain, the neuronal activity of the ventral posteromedial nucleus (VPM) of the thalamus is significantly enhanced, which may be related to the sensitization of the thalamus in inflammatory pain (Lei et al., 2024). In addition, this sensitization of the thalamus may be related to the activation of the glutamatergic neurotransmitter system and glial cells (Zhang et al., 2017). The thalamus also plays an important role in the occurrence of somatic pain. When the body is subjected to a nociceptive stimulus, pain receptors are activated and transmit signals through peripheral nerves to the spinal cord. In the spinal cord, pain signals are uploaded to the thalamus via the spinothalamic tract, which distributes the pain signals to different areas of the brain for further processing (Mendell, 2014).

All forms of chronic pain are closely associated with varying degrees of peripheral and central sensitization (Grace et al., 2021). This sensitization involves not only direct neuronal communication but also complex crosstalk among neurons, glial cells, and immune cells (McMahon et al., 2015). Regulatory factors released by immune cells play a key role in modulating nociceptor activity and pain sensitivity (Pinho-Ribeiro et al., 2017). Furthermore, the thalamus is reported to be involved in the function of many pro-inflammatory cytokines, such as IL-1β and TNF-ɑ, which are associated with pain-related hypersensitivity (Chamaa et al., 2016). Ultimately, changes in the inflammatory response and plasticity of the thalamus not only affect the perception of pain, but also have an impact on the emotional state and quality of life of patients (Hong et al., 2023).

Given the central role of the thalamus in pain perception and modulation, it represents a promising therapeutic target for chronic pain management. Although we have a preliminary understanding of the role of the thalamus in chronic pain, its precise molecular mechanisms remain largely elusive. Investigating the specific mechanisms by which different etiologies lead to the same outcome can improve our understanding of the molecular mechanisms of their complete pathways. In this study, GSE138024 and GSE222926 datasets were retrieved from the Gene Expression Omnibus (GEO) database. Bioinformatics analysis was performed to identify the common and differentially expressed genes (DEGs) in the thalamus between orofacial pain induced by injection of complete Freund’s adjuvant (CFA) into the temporomandibular joints and somatic pain induced by injection of CFA into the skin of the hind paws of the rats, followed by functional and pathway enrichment analysis. In addition, a protein–protein interaction (PPI) network was constructed, and hub genes were identified using the Search Tool for the Retrieval of Interacting Genes/Proteins (STRING) database and Cytoscape software. Finally, identical animal models were established for experimental validation to assess the reliability of our bioinformatic findings.

Although the raw transcriptomic datasets of GSE138024 and GSE222926 are publicly available, a systematic comparison of thalamic DEGs between orofacial and somatic inflammatory pain models has not yet been performed. Here, we established a comparative bioinformatics framework to map the common and pain-type-specific immune signatures in the thalamus, focusing heavily on the functional networks governing neuroinflammatory crosstalk. Crucially, the biological relevance of our computational screening was verified through experimental RT-qPCR analysis of four core overlapping genes in our laboratory’s rodent pain models. Finally, we identified distinct hub gene networks that characterize and distinguish these two distinct pain modalities.

## 2 Methods

### 2.1 Data acquisition

Our data were derived from the GEO public database on the National Center for Biotechnology Information (NCBI) website,^1^ in which the keyword retrieval “pain” selected the GSE222926 and GSE138024 high-throughput sequencing datasets. Six samples of GSE222926 were selected (Experimental group GSM6935414, GSM6935415, and GSM6935416; Control group GSM6935411, GSM6935412, and GSM6935413). The experimental group was injected with CFA into the temporomandibular joint cavity of adult rats for 14 days to induce chronic oral and facial inflammation, and the control group was injected with normal saline under the same conditions. Eight samples of GSE138024 were selected (experimental group GSM4096891, GSM4096892, GSM4096893, and GSM4096894; Control group GSM4096883, GSM4096884, GSM4096885, and GSM4096886), the experimental group was injected with CFA into the bottom of the hind paws of adult mice for 18 days to induce chronic inflammatory pain, and the control group was injected with normal saline under the same conditions.

### 2.2 Data processing and screening of DEGs

Since the two datasets originated from rats and mice, we used the “homologene” package of RStudio software, based on NCBI’s HomoloGene database, to replace the gene symbols in the mice dataset GSE138024 with their corresponding homologous genes in rats. Only genes with unambiguous one-to-one ortholog assignments were retained for subsequent analyses. According to the annotation information in the original data, the probes were converted into corresponding gene symbols, and RStudio software was used to preprocess the data. Multiple probes corresponding to a gene were averaged, genes were combined, and standardization and normalization were carried out. Principal component analysis is used to assess the biological variability between different samples. The “Limma” package in RStudio software was then used for differential analysis of gene expression. To ensure the rigor of the statistical analysis, we adopted a two-stage strategy for screening core overlapping genes. In the first stage, to maximize the detection sensitivity while avoiding the deletion of potentially relevant genes due to cross-species heterogeneity and the limited sample size of the public dataset, these genes were identified as potential DEGs for preliminary screening based on the criteria of *p* value < 0.05 and |logFC| > 1. In the second stage, in order to prioritize the identification of the genes with the most significant changes, we further applied the Benjamini-Hochberg (BH) correction method to control the false discovery rate (FDR) within 5%. The screening was conducted based on the criteria of Adj. P. Value < 0.05 and |logFC| > 1. The volcano plot visualization of DEGs was mapped in the Package “ggplot2” of R software program. Then, the Evenn tool^2^ was used to calculate the intersection of the results from the second-stage DEGs analysis of the two datasets, thereby obtaining the core overlapping DEGs. The Heatmap visualization of DEGs within the intersection was mapped in the Package “pheatmap” of the R software program.

### 2.3 Functional and pathway enrichment analysis

The overlapping DEGs of the GSE222926 and GSE138024 datasets, and the DEGs outside their respective overlapped parts, were uploaded to The Database for Annotation, Visualization and Integrated Discovery (DAVID) Gene annotation tool^3^ proceeded DEGs Gene Ontology (GO) Gene function annotation enrichment analysis and Kyoto Encyclopedia of Genes and Genomes (KEGG) pathway enrichment analysis, a *p* < 0.05 was set as the critical value.

### 2.4 PPI network construction and selection of hub genes

To explore the relationships within relevant proteins, including direct binding interactions and interconnected regulatory pathways both upstream and downstream, the STRING (version 12.0)^4^ database was utilized to construct a PPI network. Interactions with a cumulative score of more than 0.4 were deemed to have statistical significance. For visualizing this PPI network, we employed Cytoscape 3.10.1.^5^ The degree algorithm in Cytoscape’s plugin cytohubba was then used to identify the top 10 hub genes.

### 2.5 Animals

Adult male Sprague–Dawley rats (weighing 200–220 g) were randomly divided into the CTR (control) group and the CFA group. Rats were housed at 23 ± 1 °C under a 12 h light/dark cycle and had ad libitum access to standard food and water throughout the study. Prior to any experiments, all animals were allowed to acclimate to the environment for at least 7 days. After the behavioral tests, rats were anesthetized with pentobarbital sodium (50 mg/kg, i.p.). Following confirmation of deep anesthesia, animals were euthanized by an intraperitoneal overdose of pentobarbital sodium (150 mg/kg) before tissue harvesting. After collecting the samples, the death of the animal was confirmed by observing the cessation of its heartbeat and breathing.

### 2.6 Temporomandibular joint inflammation and somatic inflammatory pain model

We anesthetized the rats by intraperitoneal injection of a 50 mg/kg dose of pentobarbital sodium. At the point one-third away from the line connecting the ears and eyes of the rat, we used an insulin syringe to inject 50 μL of CFA (Sigma-Aldrich, Saint Louis, United States) into the bilateral temporomandibular joint cavities of the rat. Rats in the CTR group were injected bilaterally with 50 μL 0.9% saline (*n* = 5 per group). The model of somatic inflammatory pain was constructed using a group of independent laboratory rats through the same method. CFA was injected into the skin of the bilateral hind paws of rats, while the CTR group was injected with 0.9% saline (n = 5 per group). In order to ensure that the duration of inflammatory pain matches the time period of the model in the dataset, a second identical injection of CFA or saline was administered to the respective groups on day 8.

### 2.7 von-Frey filaments test

A total of 1 day before the injection and on the 1st, 3rd, 5th, and 7th days after injection of CFA or saline, the rats were placed in the test environment to acclimate for 1 h. von-Frey filaments were applied to the upper part of the rat’s temporomandibular joint cavity or to the skin of the footpad of the injection area until a bending occurred. The force applied by the filaments was gradually increased (0.008–10 g) until a withdrawal response of the head or footpad was triggered. Each filament was tested at intervals of 30 s, and a total of five tests were conducted. If an escape behavior was observed at least three times after using the filament for exploration, the rat was considered to have a positive reaction to that filament. The threshold of the reaction was defined as the minimum force value that triggered the withdrawal response at least three times during the five tests (Fruhstorfer et al., 2001). Finally, the changes in the threshold values of each group of rats were statistically analyzed to determine the successful establishment of the model.

### 2.8 Modified safranin O and fast green staining

The temporomandibular joints were collected on day 14 after CFA or saline injection. The tissues were fixed in 4% paraformaldehyde, decalcified in EDTA decalcifying solution (Solarbio, Beijing, China) for about 1 month, dehydrated under the 100, 95, 90, and 80% ethanol concentration gradient, and the cartilage was embedded in paraffin. The specimen was then cut into 5 μm sections. Paraffin section staining was performed using a Modified Safranin O and Fast Green Stain Kit (Solarbio, Beijing, China). We observed the changes in the cartilage matrix of the temporomandibular joint to determine the level of inflammation.

### 2.9 Reverse transcription-quantitative polymerase chain reaction

The thalamic tissues from the orofacial inflammatory pain model were collected on day 14 after injection of CFA or saline, and the thalamic tissues from the somatic inflammatory pain model were collected on day 18 after injection of CFA or saline, which corresponds to the time point of the transcriptomic datasets. The mRNA expression levels of the DEGs C-C motif chemokine receptor 1 (*Ccr1*), C-X-C motif chemokine ligand 13 (*Cxcl13*), lipocalin 2 (*Lcn2*), S100 calcium binding protein A8 (*S100a8*), and C-C motif chemokine ligand 2 (*Ccl2*) in the rat thalamus were detected by RT-qPCR. Since *Cxcl13, Lcn2,* and *Ccl2* are core overlapping genes, *Ccr1* ranks high in the PPI network analysis; *S100a8* and its functional annotations (inflammatory response, chemotaxis, cytokine activity, and IL-17 signaling pathway) are directly related to our neuroimmune hypothesis. Therefore, we selected them for verification based on these criteria, allowing us to validate candidate genes that are both statistically reliable and biologically reasonable. Total RNA isolations were carried out using the Animal Tissue Total RNA Extraction Kit (Servicebio, Wuhan, China) according to the manufacturer’s instructions. The total RNA was converted to cDNA using PrimeScriptTM RT Master Mix (Takara Bio, Kyoto, Japan), and RT-qPCR was conducted using TB Green^®^ Premix Ex TaqTM II (Takara Bio, Kyoto, Japan). GAPDH was used as internal control. The sequences of primers are given in Table 1.

**Table 1:** Sequences of the primers for RT-qPCR.

Gene	Forward primer (5′–3′)	Reverse primer (5′–3′)
*Ccr1*	TTGCCTACACCCACTGTTGT	GCGGTATAGCCACATGCCTT
*Cxcl13*	CAAGTTATACGCCCTGGGAATG	CCATCTGGCAGTAGGATTCACAC
*Lcn2*	ACAACGTCACTTCCATCCTCGT	TGGCAAACTGGTCGTAGTCAGT
*S100a8*	GTGCCCTCAGTTTGTGCAGAATA	TTACTCCTTFTGGCTGTCTTTATG
*Ccl2*	CTCAAGAACATCCAAAGTGTG	ATTCTTGAGTGTGGCTATGAC
*Gapdh*	GACATGCCGCCTGGAGAAAC	AGCCCAGGATGCCCTTTAGT

### 2.10 Western blot

To verify protein-level expression of candidate genes, a Western blot was performed on thalamic tissues from rats in both inflammatory pain models and their respective CTR groups. Total protein was extracted using RIPA lysis buffer (Solarbio, Beijing, China) supplemented with protease and phosphatase inhibitors. Protein concentrations were determined by BCA assay (Thermo Fisher Scientific, Waltham, MA, United States). Equal amounts of protein (30 μg) were separated by SDS-PAGE and transferred onto PVDF membranes (Millipore, Billerica, MA, United States). Membranes were blocked with 5% non-fat milk in TBST for 2 h at room temperature, then incubated overnight at 4 °C with primary antibodies against CCR1, CXCL13, and GAPDH (as loading control). After washing, membranes were incubated with HRP-conjugated secondary antibodies for 2 h. Protein bands were visualized using an ECL detection kit and imaged with a ChemiDoc™ system. Densitometric analysis was performed using ImageJ software, with target protein expression normalized to GAPDH.

### 2.11 Statistical analysis

GraphPad Prism 9.5.1 software was used for statistical analysis. All data were presented as mean ± SEM, and *p* < 0.05 were considered statistically significant. For RT-qPCR and Western blot data that did not meet the assumptions of normality or homogeneity of variance, group comparisons were conducted using the two-tailed Mann–Whitney U test. Two-way repeated-measures analysis of variance (ANOVA) followed by Bonferroni’s *post-hoc* test was used for mechanical pain threshold test analysis.

## 3 Results

### 3.1 Screening of DEGs

**Figure 1:**
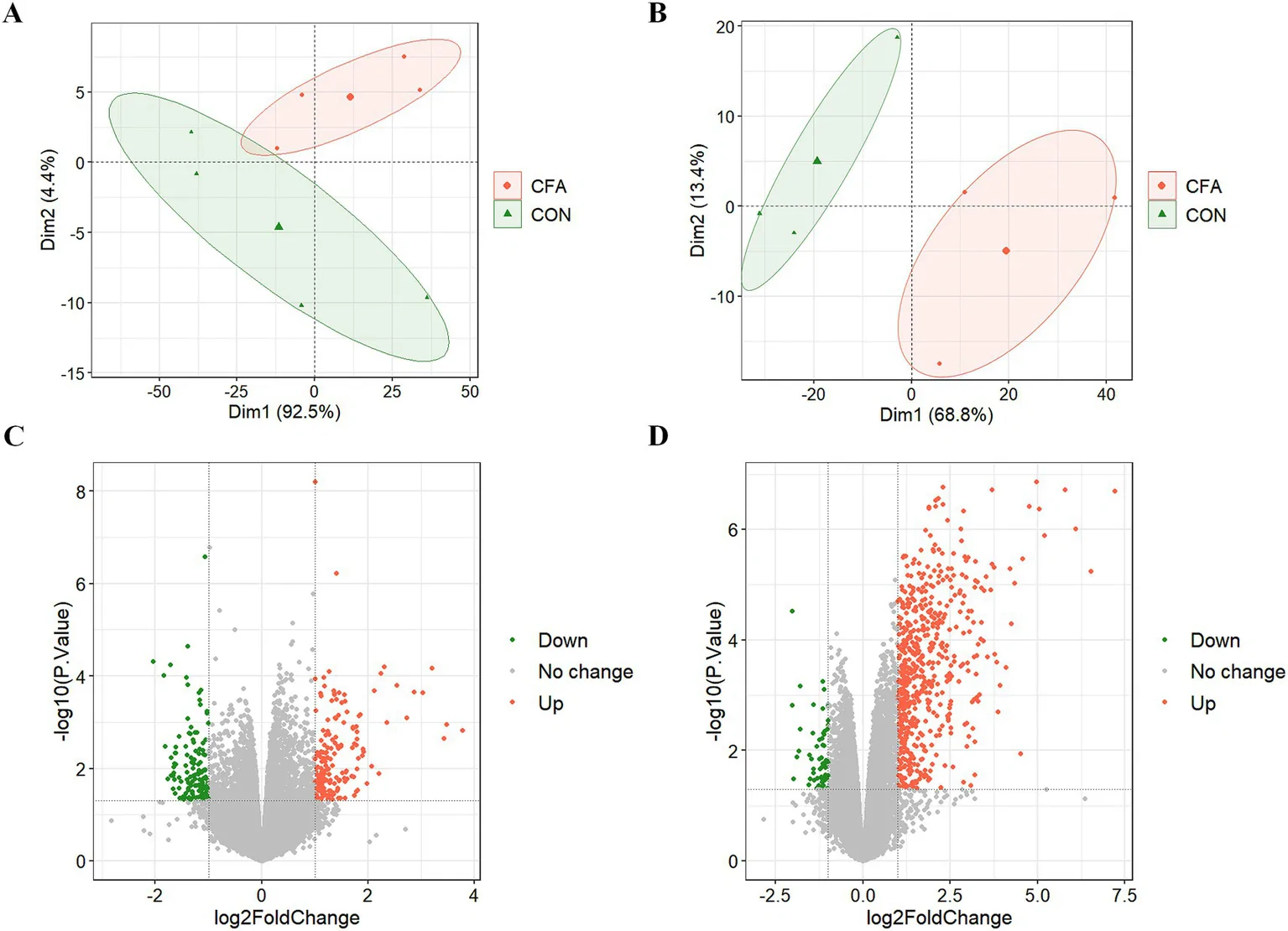
Analysis of differentially expressed genes (DEGs). **(A)** Principal component analysis of GSE138024. **(B)** Principal component analysis of GSE222926. **(C)** Volcano plot depicting the DEGs in GSE138024. **(D)** Volcano plot depicting the DEGs in GSE222926.

After data normalization, principal component analysis indicated the two principal component scores for Dimension 1 and Dimension 2 of GSE138024 were 92.5 and 4.4%, respectively, indicating a significant difference between the Control and CFA group samples. The PCA plot demonstrated a clear segregation between the control and CFA groups, with tight intra-group clustering (Figure 1A). The two principal component scores for Dimension 1 and Dimension 2 of GSE222926 were 68.8 and 13.4%, respectively, indicating a significant difference between the Control and CFA group samples, with gene expression changing to a large extent after CFA model establishment (Figure 1B). Then the gene expression difference was analyzed; a total of 326 DEGs were screened in GSE138024, comprising 191 upregulated and 135 downregulated genes (Figure 1C). A total of 657 DEGs were screened in GSE222926, of which 592 genes were upregulated, and 65 genes were downregulated (Figure 1D).

### 3.2 Bioinformatics analysis of the overlapping DEGs

**Figure 2:**
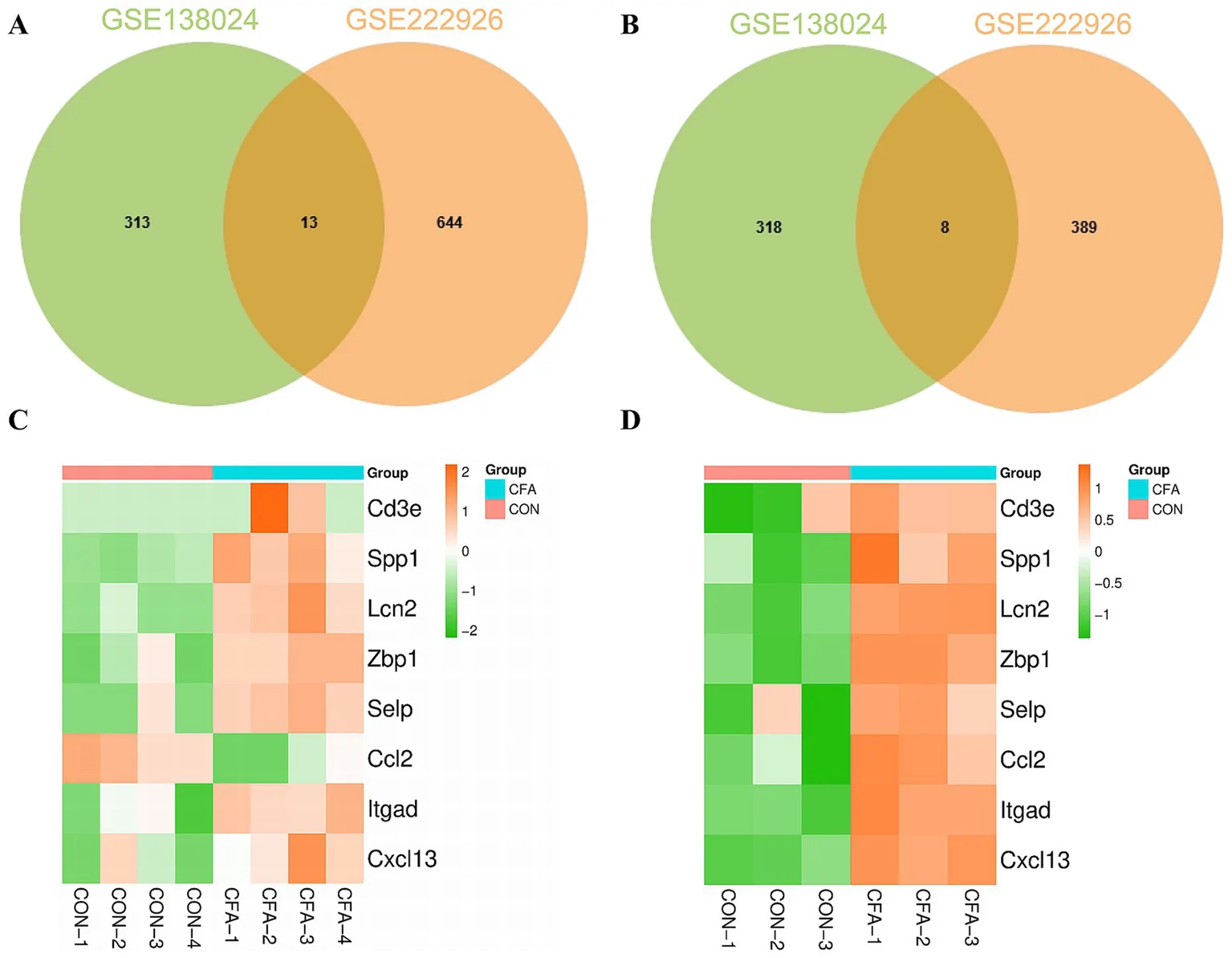
Analysis of the overlapping differentially expressed genes (DEGs). **(A)** The intersection number of DEGs in the first DEGs analysis stage of GSE138024 and GSE222926. **(B)** The intersection number of DEGs in the second DEGs analysis stage of GSE138024 and GSE222926. **(C)** Heatmap of the overlapping DEGs in GSE138024. **(D)** Heatmap of the overlapping DEGs in GSE222926.

After the DEGs analysis in the first stage, by overlapping the DEGs of GSE138024 and GSE222926 through Evenn, 13 common DEGs were obtained: cocaine- and amphetamine-regulated transcript prepropeptide (*Cartpt*), periostin, osteoblast specific factor (*Postn*), *Ccr1*, CD3 antigen, epsilon polypeptide (*Cd3e*), secreted phosphoprotein 1 (*Spp1*), *Lcn2*, Z-DNA binding protein 1 (*Zbp1*), *S100a8*, selectin P (*Selp*), *Ccl2*, integrin subunit alpha D (*Itgad*), ubiquitin D (*Ubd*), and *Cxcl13* (Figure 2A). After the DEGs analysis in the second stage, by overlapping the DEGs of GSE138024 and GSE222926 through Evenn, eight common DEGs were obtained: *Cd3e*, *Spp1*, *Lcn2*, *Zbp1*, *Selp*, *Ccl2*, *Itgad*, and *Cxcl13* (Figure 2B), These are the core overlapping genes. The expression and information of the core overlapping DEGs in the two datasets are shown in Table 2, where the gene *Ccl2* was downregulated in GSE138024, but upregulated in GSE222926. In addition, the RStudio software package “pheatmap” was used to draw the expression heatmap of these eight8 core overlapping genes in GSE138024 and GSE222926 (Figures 2C,D).

**Table 2:** Expression details of the core overlapping differentially expressed genes (DEGs).

Symbol	LogFC	*p*-value	Group	Description
GSE138024				
*Cd3e*	1.3715812	0.000792	Up	CD3 epsilon subunit of T-cell receptor complex
*Spp1*	1.3940208	0.0008185	Up	Secreted phosphoprotein 1
*Lcn2*	2.3447422	0.0010058	Up	Lipocalin 2
*Zbp1*	1.7161662	0.0012853	Up	Z-DNA binding protein 1
*Selp*	1.9124707	0.0043219	Up	Selectin P
*Ccl2*	−1.333938	0.0056961	Down	C-C motif chemokine ligand 2
*Itgad*	1.1919778	0.0069372	Up	Integrin subunit alpha D
*Cxcl13*	1.5618324	0.0435414	Up	C-X-C motif chemokine ligand 13
GSE222926				
*Cd3e*	1.0879972	0.0001465	Up	CD3 epsilon subunit of T-cell receptor complex
*Spp1*	3.3305173	0.0009618	Up	secreted phosphoprotein 1
*Lcn2*	7.2148193	0.0000002	Up	lipocalin 2
*Zbp1*	1.1372839	0.0000251	Up	Z-DNA binding protein 1
*Selp*	1.4459269	0.0012743	Up	Selectin P
*Ccl2*	3.8286554	0.000252	Up	C-C motif chemokine ligand 2
*Itgad*	1.7659066	0.0000183	Up	Integrin subunit alpha D
*Cxcl13*	6.0846651	0.0000009	Up	C-X-C motif chemokine ligand 13

### 3.3 Functional and pathway enrichment analysis of DEGs

**Figure 3:**
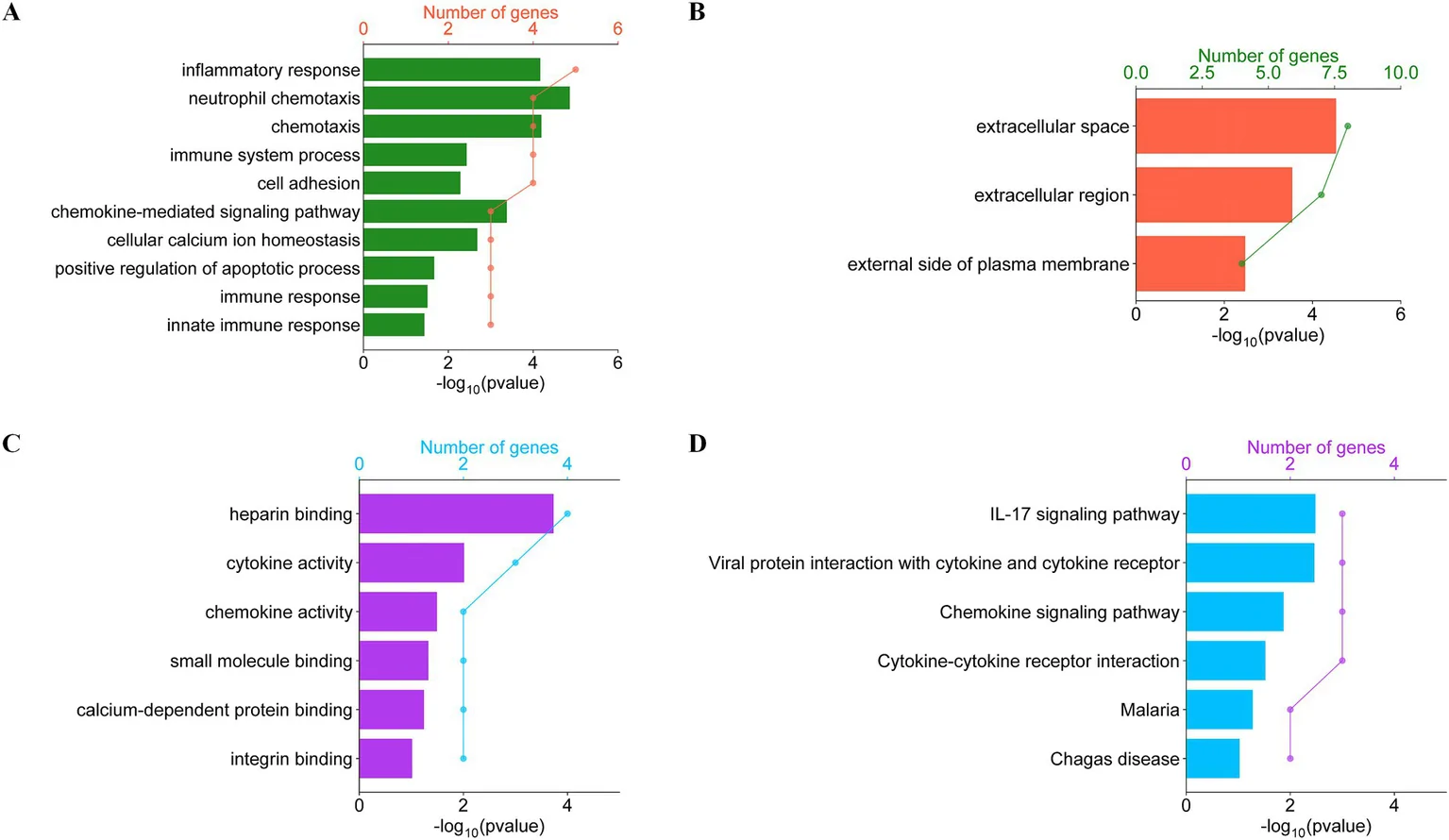
GO analysis and KEGG pathway analysis of the overlapping differentially expressed genes (DEGs). **(A)** Enriched biological processes (BP) identified in GO analysis. **(B)** Cellular component (CC) terms enriched in GO analysis. **(C)** Molecular function (MF) terms enriched in GO analysis. **(D)** KEGG pathway enrichment analysis.

To study the biological functions and pathways associated with the overlapping DEGs, 313 non-overlapping DEGs of GSE138024 and 644 non-overlapping DEGs of GSE222926, GO and KEGG pathway enrichment analyses were performed. The results of GO analysis showed that the biological process of gene enrichment of the overlapping DEGs was mainly related to inflammatory response, neutrophil chemotaxis, chemotaxis, and immune system process (Figure 3A). Cell components were mainly enriched in the extracellular space, extracellular region, and external side of the plasma membrane (Figure 3B). Molecular functions were mainly related to heparin binding, cytokine activity, chemokine activity, small molecule binding, and calcium-dependent protein binding (Figure 3C). KEGG pathway analysis showed that the IL-17 signaling pathway, viral protein interaction with cytokine and cytokine receptor, and the Chemokine signaling pathway are significantly enriched (Figure 3D). The specific information and gene distribution of the overlapping DEGs enrichment analysis were shown in Table 3.

**Table 3:** Specific information on the overlapping differentially expressed genes (DEGs) enrichment analysis.

Term	Count	*P*-value	Genes
BP			
Inflammatory response	5	0.0000681	*Ccr1, Selp, Ccl2, Cxcl13, S100a8*
Neutrophil chemotaxis	4	0.0000138	*Spp1, Ccl2, Cxcl13, S100a8*
Chemotaxis	4	0.0000642	*Ccr1, Ccl2, Cxcl13, S100a8*
Immune system process	4	0.0037143	*Zbp1, Lcn2, Cd3e, S100a8*
Cell adhesion	4	0.0052166	*Selp, Postn, Itgad, Spp1*
CC			
Extracellular space	8	0.0000295	*Selp, Postn, Lcn2, Spp1, Ccl2, Cartpt, Cxcl13, S100a8*
Extracellular region	7	0.000288	*Postn, Lcn2, Spp1, Ccl2, Cartpt, Cxcl13, S100a8*
External side of plasma Membrane	4	0.0033606	*Ccr1, Selp, Itgad, Cd3e*
MF			
Heparin binding	4	0.000186	*Selp, Postn, Ccl2, Cxcl13*
Cytokine activity	3	0.0097636	*Spp1, Ccl2, Cxcl13*
Chemokine activity	2	0.0322094	*Ccl2, Cxcl13*
Small molecule binding	2	0.0470352	*Lcn2, Spp1*
Calcium-dependent protein binding	2	0.0574105	*Selp, S100a8*
KEGG			
IL-17 signaling pathway	3	0.0032888	*Lcn2, Ccl2, S100a8*
Viral protein interaction with cytokine and Cytokine receptor	3	0.0034292	*Ccr1, Ccl2, Cxcl13*
Chemokine signaling pathway	3	0.0134230	*Ccr1, Ccl2, Cxcl13*
Cytokine-cytokine receptor	3	0.0299773	*Ccr1, Ccl2, Cxcl13*
Interaction malaria	2	0.0529450	*Selp, Ccl2*

**Figure 4:**
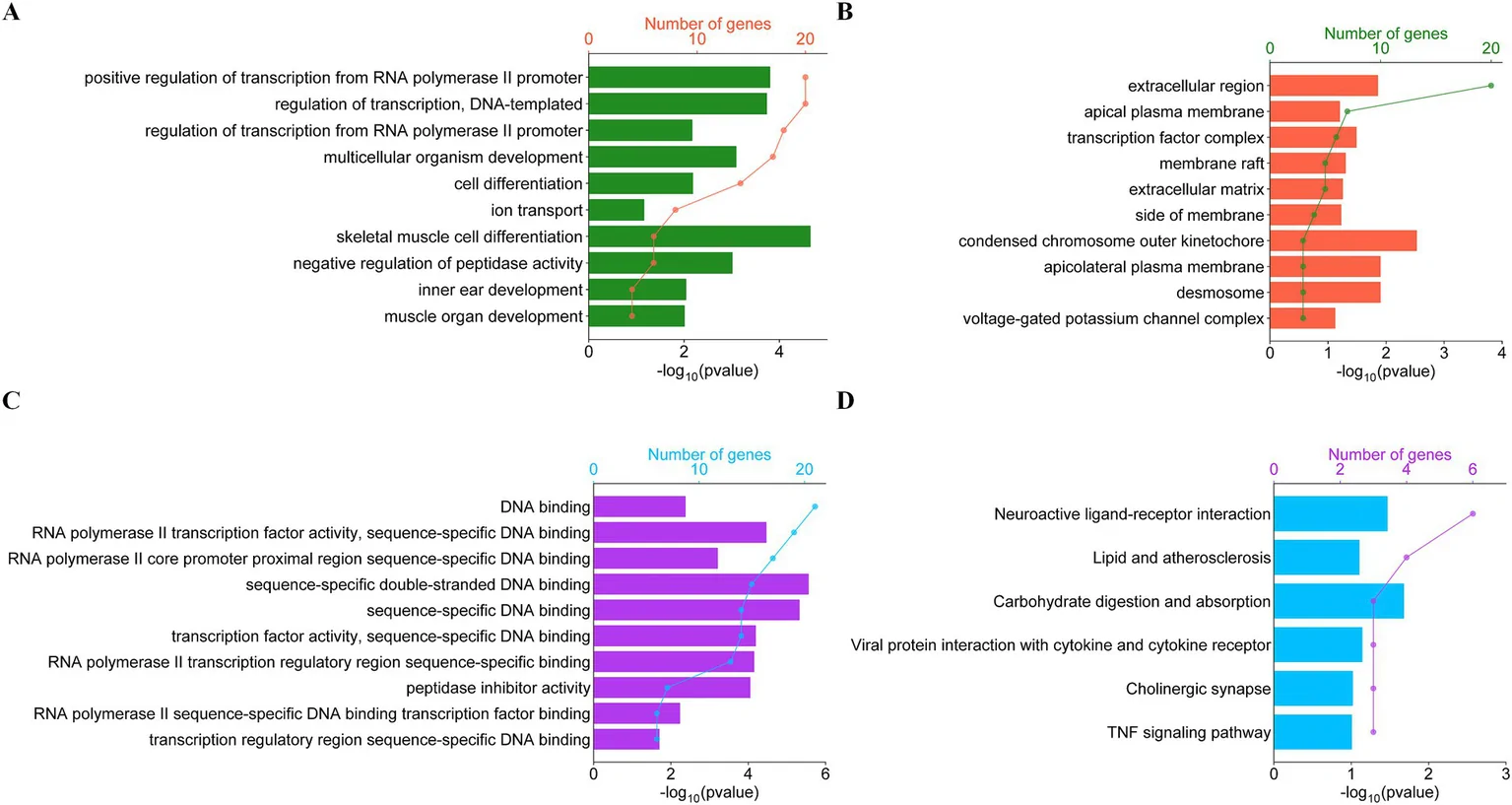
GO analysis and KEGG pathway analysis of parts outside the overlapping differentially expressed genes (DEGs) in GSE138024 **(A)** Enriched biological processes (BP) identified in GO analysis. **(B)** Cellular component (CC) terms enriched in GO analysis. **(C)** Molecular function (MF) terms enriched in GO analysis. **(D)** KEGG pathway enrichment analysis.

GO analysis of non-overlapping DEGs of GSE138024 showed that the biological processes were mainly enriched with positive regulation of transcription from the RNA polymerase II promoter and regulation of transcription, and DNA-templated (Figure 4A). Cell components were mainly enriched in the extracellular region, apical plasma membrane (Figure 4B). Molecular functions were mainly related to DNA binding, RNA polymerase II transcription factor activity, and sequence-specific DNA binding (Figure 4C). KEGG pathway analysis showed that neuroactive ligand-receptor interaction, lipids, and atherosclerosis were significantly enriched (Figure 4D).

**Figure 5:**
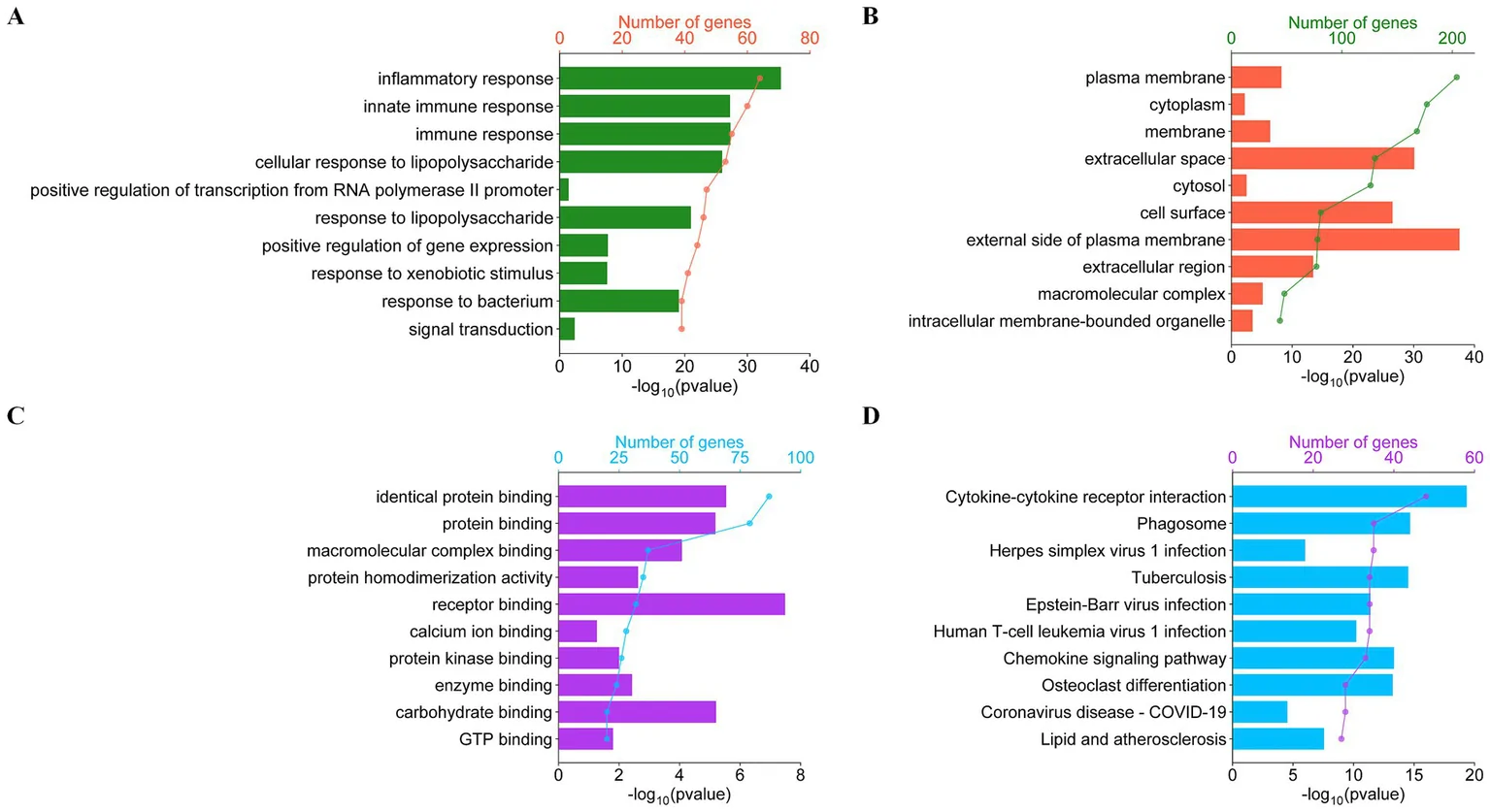
GO analysis and KEGG pathway analysis of parts outside the overlapping differentially expressed genes (DEGs) in GSE222926. **(A)** Enriched biological processes (BP) identified in GO analysis. **(B)** Cellular component (CC) terms enriched in GO analysis. **(C)** Molecular function (MF) terms enriched in GO analysis. **(D)** KEGG pathway enrichment analysis.

GO analysis of genes outside the overlapping DEGs of GSE222926 showed that the biological processes were mainly enriched with inflammatory response, innate immune response (Figure 5A); Cell components were mainly enriched in plasma membrane, cytoplasm (Figure 5B). Molecular functions were mainly related to identical protein binding (Figure 5C). KEGG pathway analysis showed that cytokine-cytokine receptor interaction and phagosomes were significantly enriched (Figure 5D).

### 3.4 Construction of PPI networks and selection of core genes

**Figure 6:**
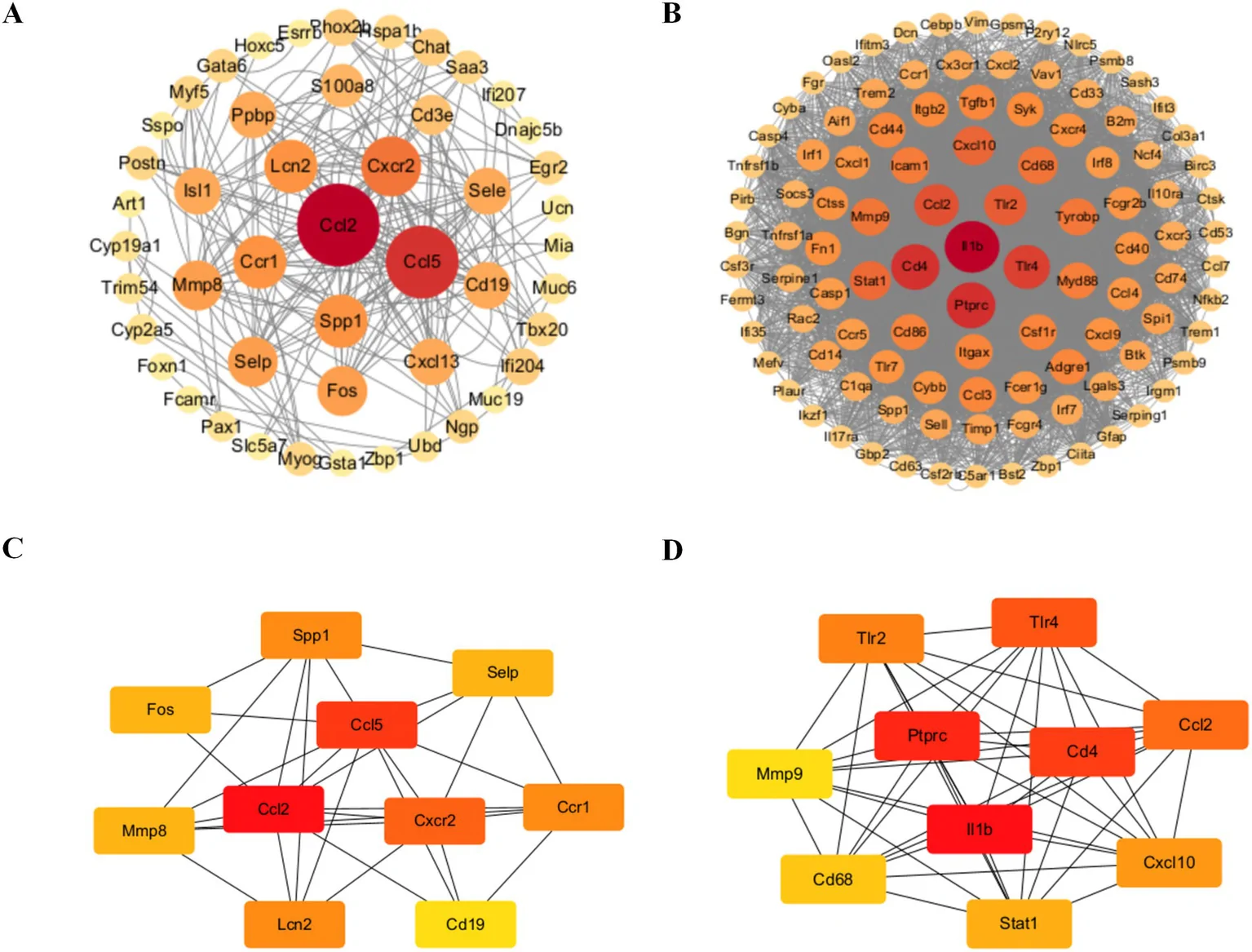
Protein–protein interaction (PPI) network map and hub gene. **(A)** PPI network map of GSE138024, PPI network map of GSE222926 **(B)**, hub genes of GSE138024 **(C)**, and hub genes of GSE222926 **(D)**.

To investigate the interactions between DEGs of GSE138024 and GSE222926, a PPI network was constructed in STRING, and interactions with a minimum required interaction score greater than 0.4 were considered statistically significant. Moreover, the network is visualized and optimized according to a degree algorithm using Cytoscape 3.10.1 software (Figures 6A,B). The top 10 hub genes were extracted using Cytoscape’s plugin cytohubba. We extracted the following hub genes from GSE138024: *Ccl2*, C-C motif chemokine ligand 5 (*Ccl5*), chemokine (C-X-C motif) receptor 2 (*Cxcr2*), *Lcn2*, *Spp1*, *Ccr1*, matrix metallopeptidase 8 (*Mmp8*), fos proto-oncogene, AP-1 transcription factor subunit (*Fos*), *Selp*, and CD19 antigen (*Cd19*) (Figure 6C); the expressions of *Ccl2* and *Fos* were downregulated, while the expressions of other genes were upregulated. We then extracted the hub genes from GSE222926: these included interleukin 1 beta (*Il1β*), protein tyrosine phosphatase receptor type C (*Ptprc*), CD4 antigen (*Cd4*), toll-like receptor 4 (*Tlr4*), *Ccl2*, toll-like receptor 2 (*Tlr2*), C-X-C motif chemokine ligand 10 (*Cxcl10*), signal transducer and activator of transcription 1 (*Stat1*), CD68 antigen (*Cd68*), and matrix metallopeptidase 9 (*Mmp9*) (Figure 6D). The expression of these hub genes was significantly upregulated. Notably, five of the overlapping DEGs (*Ccr1*, *Lcn2*, *Spp1*, *Ccl2*, and *Selp*) were successfully captured within the top 10 hubs of GSE138024, while *Ccl2* uniquely emerged as a hub gene in both distinct pain modalities.

### 3.5 Verification of partially overlapping genes

**Figure 7:**
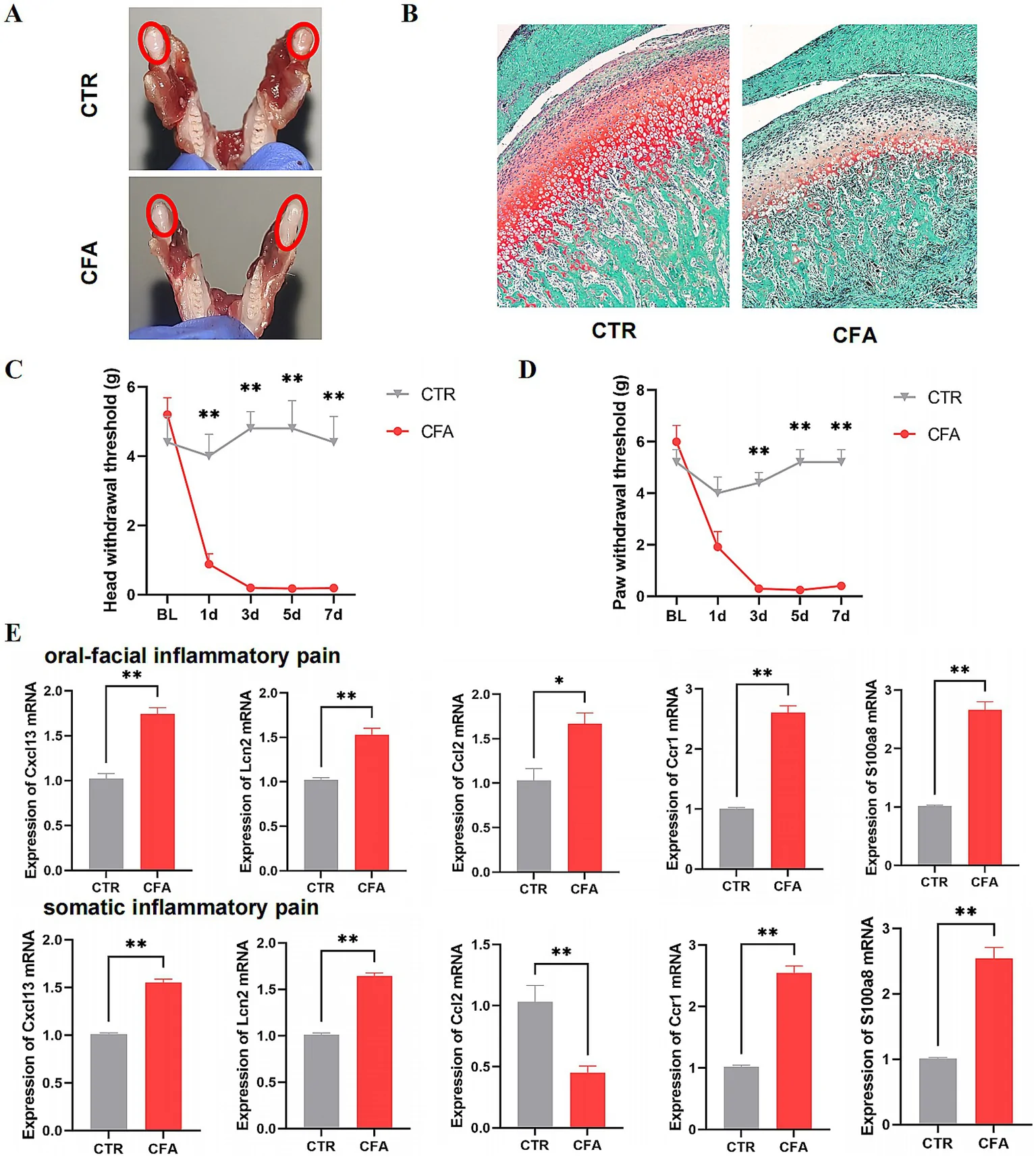
Changes in thalamic overlapping gene expression in two complete Freund’s adjuvant (CFA)-induced pain models **(A)** Comparison of the appearance and morphology of the condylar cartilage of the temporomandibular joint between the CFA group and the control (CTR) group. **(B)** Safranin O (red) and Fast Green (green) staining of cartilage, showing a marked loss of proteoglycans in the CFA group compared to the CTR group. **(C,D)** The mechanical pain threshold of the orofacial area significantly decreased from day 1 to day 7. Similarly, the paw withdrawal threshold was significantly reduced from day 3 through day 7. **(E)** RT-qPCR assay of some overlapping DEGs expression in the thalamus showed that in the thalamus of both pain models, the mRNA levels of *Ccr1*, *Cxcl13*, *Lcn2,* and *S100a8* were all increased compared with the CTR group. In contrast, the mRNA level of *Ccl2* increased in the thalamus of the orofacial inflammatory pain model and decreased in the thalamus of the somatic inflammatory pain model. All data were normalized to the Gapdh. Data represent the mean ± SEM, *n* = 5. *P < 0.05, **P < 0.01, ***P < 0.001.

By inducing temporomandibular joint inflammation and paw inflammation in rats, inflammatory facial pain and somatic pain in rats were established. From the changes in the structural morphology of the mandibular condyle and the staining results of the temporomandibular joint cartilage, it can be seen that the cartilage structure of the temporomandibular joint of the rats after CFA injection has collapsed, and the proteoglycan of cartilage has decreased, indicating that the injection of CFA successfully induced temporomandibular joint inflammation (Figures 7A,B). The von-Frey filaments test indicated that the mechanical pain thresholds for the two types of pain began to decrease after the injection of CFA and remained for 7 days (Figures 7C,D). The RT-qPCR test confirmed that the expression levels of the overlapping DEGs *Ccr1*, *Cxcl13*, *Lcn2*, *S100a8,* and *Ccl2* in the rat thalamus for both types of pain were consistent with the screening results (Figure 7E), demonstrating the reliability of the analytical data.

**Figure 8:**
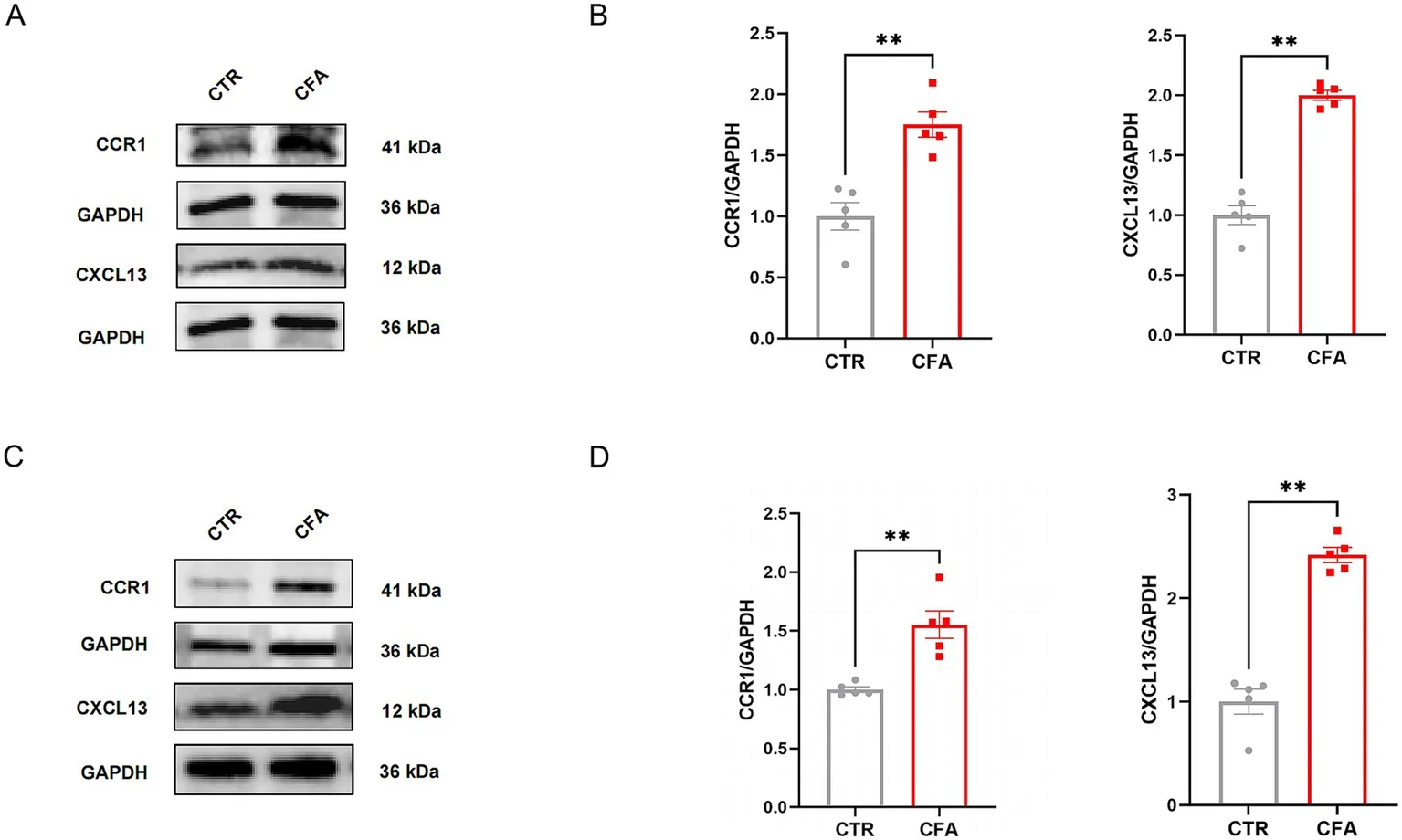
Protein expression of CCR1 and CXCL13 in the thalamus of orofacial and somatic inflammatory pain models. **(A)** Representative Western blot bands of CCR1, CXCL13, and GAPDH (loading control) in the orofacial pain model. **(B)** Densitometric quantification of CCR1 and CXCL13 protein levels normalized to GAPDH. **(C)** Representative Western blot bands of CCR1, CXCL13, and GAPDH (loading control) in the somatic pain model. **(D)** Densitometric quantification of CCR1 and CXCL13 protein levels normalized to GAPDH. Data are presented as mean ± SEM, *n* = 5. *p < 0.05, **p < 0.01.

Then, we assessed the protein levels of CCR1 and CXCL13 in thalamic tissues from two different rat models. These two targets were selected to represent complementary categories from our two-stage screening strategy: CXCL13 was identified as a core overlapping DEG under strict FDR correction, while CCR1 met the nominal screening criteria and showed strong biological relevance (hub gene in PPI network, involvement in chemokine signaling) despite not surviving FDR correction. This selection allowed us to validate both statistically robust hits and biology-driven candidates at the protein level. As shown in Figure 8, protein expression levels of both CCR1 and CXCL13 were significantly elevated in the CFA groups compared with their respective CTR groups in both models. These results are consistent with our RT-qPCR findings and confirm that the observed transcriptional upregulation of these genes translates to increased protein expression in the thalamus during chronic inflammatory pain.

## 4 Discussion

As a key relay station in the brain, the thalamus plays a crucial role in the transmission and processing of pain signals. The thalamus receives sensory information from various parts of the body and transmits this information to other areas of the brain, including the sensory cortex and brain regions associated with emotional processing, such as the anterior cingulate cortex (ACC) and the amygdala. The dorso-medial thalamic nucleus (MD) was found to specifically encode pain information, and its connection to the anterior cingulate cortex plays a role in pain processing (Tu et al., 2024). In addition, studies on thalamic-specific pain field potential response signals have better understood the dynamic characteristics of the thalamus in pain information processing (Li et al., 2024). The study of the relationship between pain and the thalamus not only contributes to a deeper understanding of the neurobiological basis of pain but may also facilitate the development of targeted interventions, particularly for the treatment of chronic pain and pain comorbidities.

CFA is an adjuvant commonly used to elicit inflammatory pain in animal models, capable of provoking a sustained inflammatory response and pain sensitization, simulating chronic inflammatory pain in humans (Cui et al., 2024). Consequently, CFA-induced inflammatory pain models are widely used to study the mechanism of pain and evaluate potential analgesic agents (Ma et al., 2022; Zhang et al., 2022). In this study, our analysis reveals potential targets for mechanisms of changes in the role of orofacial pain and somatic pain in the thalamus. In these two inflammatory pain models, 13 DEGs were identified by bioinformatics analysis: *Cartpt*, *Postn*, *Ccr1*, *Cd3e*, *Spp1*, *Lcn2*, *Zbp1*, *S100a8*, *Selp*, *Ccl2*, *Itgad*, *Ubd,* and *Cxcl13*. Among them, *Cartpt*, *Ccl2*, and *Ubd* were upregulated in GSE222926 but downregulated in GSE138024, while other genes were upregulated in both datasets. This suggests that the two pain models share common molecular mechanisms, but also possess their own unique characteristics. Although the results were derived from cross-species analysis, after homologous gene normalization processing, these results are of great significance for identifying the common immune-related changes in the thalamus for different types of pain, thereby providing a direction for future pain intervention research.

Overlapping genes *Cartpt*, *Spp1*, *Ccl2*, and *Cxcl13* are closely associated with neuropathic pain. Cartpt play a role in appetite, energy balance, weight maintenance, reward and addiction, and stress response (Ahmadian-Moghadam et al., 2018), and they are associated with hypersensitivity and neuropathic pain (Yosten et al., 2020). Spp1, which encodes a protein involved in the attachment of osteoclasts to the mineralized bone matrix, is also a cytokine that upregulates IFN-*γ* and IL-12 expression. Studies have shown that Spp1 shRNA enhances microglial proliferation and apoptosis, inhibits microglial death, improves pain perception, and inhibits microglial activation in rats with selective neuralgia (Chen et al., 2024). SPP1 is enriched in biological functions of neutrophil chemotaxis, extracellular space, extracellular region, cytokine activity, and small molecule binding. CXCL13 is an antimicrobial peptide and CXC a chemoattractant that is strongly expressed in the splenic follicles and lymph nodes. Based on enrichment analysis of biological functions and pathways, Cxcl13 is enriched in the inflammatory response, neutrophil chemotaxis, chemotaxis, extracellular space, extracellular region, heparin binding, cytokine activity, chemokine activity, viral protein interaction with cytokine and cytokine receptors, the chemokine signaling pathway, and the cytokine–cytokine receptor interaction pathway. Inhibition of CXCL13 in the mouse spinal cord, or deletion of its receptor Cxcr5, significantly reduced mechanically abnormal pain in a mouse model of complex regional pain syndrome type I (CRPS-I), as well as hyperactivation of spinal glial cells and activation of c-Fos (Wang et al., 2023). The cis-acting silencer identified in the distal upstream of the Cxcl13 promoter inhibits Cxcl13 transcription by binding to zinc finger protein 382 (ZNF382) in neurons. Blocking this binding or gene deletion of this silencing agent removes ZNF382’s inhibition of Cxcl13 transcription and inhibits ZNF382-induced anti-nociception, thereby reducing neuropathic pain (Ma et al., 2021). CCL2 chemokines are a superfamily of secreted proteins involved in neuroimmune communication in pain involved in both peripheral and central pain sensitization. Products of the Ccl2 gene play a role in a variety of biological processes, including cell migration, inflammatory response, and tumor progression. By binding to its specific receptor CCR2, CCL2 activates downstream signaling pathways, such as JAK/STAT, p38/MAPK, and PI3K/AKT, which regulate multiple transcription factors and genes, leading to cell growth, proliferation, migration, and increased CCL2 expression (Xu et al., 2021). CXCL1 and CCL2 drive pain via neuronal CXCR2 and astrocyte receptors. In models of inflammation and neuropathy, CXCL13, CCL21, and CX3CL1 also promote central sensitization by activating spinal cord astrocytes and microglia, leading to allodynia and hyperalgesia (Qu et al., 2024). Moreover, our analyses show that Ccl2 is enriched in the inflammatory response, neutrophil chemotaxis, extracellular space, extracellular region, heparin binding, cytokine activity, chemokine activity, and the IL-17 signaling pathway. Some studies have analyzed that chronic constriction injury (CCI) causes increased expression of CCL2 in the dorsal root ganglion (DRG) of neuropathic pain, indicating that CCL2 may also be related to hypersensitivity and neuropathic pain (Kwiatkowski et al., 2017).

Accumulating evidence indicates that *Ccr1*, *Cd3e*, *Lcn2*, *Zbp1*, *Itgad*, *Ubd*, and *S100a8* genes are involved in immune and inflammatory regulatory processes. Ccr1 is a gene encoding a member of the β-chemokine receptor family, and ligands for CCR1, including macrophage inflammatory protein 1ɑ (MIP-1ɑ), which is regulated by normal T expression and activation of secreted proteins (RANTES) (Furuichi et al., 2000). Signal transduction mediated by chemokines and their receptors is essential for effector immune cells to recruit to the site of inflammation. Endogenous ligands of CCR1 are involved in the development of neuropathic pain (CCL2/3/5/7/8/9) and the persistence of pain (CCL2/7/8) (Pawlik et al., 2022). Cd3e, which encodes a protein called CD3-*ε* polypeptide, together with CD3-*γ*, −*δ*, and -*ζ*, and T cell receptor ɑ/β and γ/δ heterodimers, forms the T cell receptor-CD3 complex, which plays an important role in coupling antigen recognition with several intracellular signal transduction pathways (Wu et al., 2020). Lcn2 encodes a protein that belongs to the lipid carrier protein family, which is responsible for transporting hydrophobic small molecules, including lipids, steroid hormones, and retinoids. LCN2 is a neutrophil gelatinase-associated lipid carrier protein, and it plays a role in innate immunity by isolating ferriferous carriers that limit bacterial growth. Lcn2 is enriched in the biological functions of the immune system process, extracellular space, extracellular region, and small molecule binding. Astrocyte endoplasmic reticulum stress sensors PERK (PKR-like ER Kinase) and IRE1 (Inositol-Requiring Enzyme 1) have been shown to promote morphine tolerance and hyperalgesia by upregulating LCN2 and NLRP3 in the spinal cord (Wang et al., 2024). Zbp1 plays a role in the innate immune response by binding to foreign DNA and inducing type I interferon production, as a target of IRF3 (interferon regulatory factor 3) associated with various neuroinflammatory diseases, such as Alzheimer’s disease (Joshi et al., 2024). Itgad, a gene belonging to the β-2 integrin family of membrane glycoproteins, encodes the alpha subunit of the heterodimer on the cell surface. It is expressed in tissue and circulating myeloid white blood cells, and it may have important functions in clinical syndromes such as infection, inflammation, and tissue damage (Miyazaki et al., 2014). UBD is a member of the ubiquitin-like modification family and appears to have a similar function to ubiquitin. Studies have shown that UBD may mediate the activation of p38 MAPK, thereby promoting the proliferation of Rheumatoid Arthritis Fibroblast-Like Synoviocytes (RA-FLSs) and ultimately the progression of rheumatoid arthritis (RA) (Chen et al., 2023).

S100a8 encodes a protein that is a member of the S100 protein family and contains two EF hand calcium-binding motifs. The S100 protein is involved in the regulation of many cellular processes, such as cell cycle progression and differentiation. Enrichment analysis based on function and pathway shows that S100a8 is enriched in biological functions with the inflammatory response, neutrophil chemotaxis, immune system process, extracellular space, extracellular region, calcium-dependent protein binding, and in IL-17 signaling pathway. The study found that interference with S100a8 in microglia increased the pain threshold during migraine attacks and inhibited neuroinflammation and microglia activation (An et al., 2024). S100a8 is used as a potential biomarker for fibromyalgia (FM), in the pathophysiology of fibromyalgia may be mediated by Receptor for Advanced Glycation Endproducts (RAGE) and TLR4, activates Activator Protein-1 (AP-1) and Nuclear Factor kappa-light-chain-enhancer of activated B cells (NF-κB). This signaling triggers the release of pro-inflammatory cytokines, thereby contributing to the characteristic inflammation, fatigue, and chronic pain of fibromyalgia (Vega-Ramírez et al., 2024). Intradermal injections of S100A8/A9 enhanced itch-related behaviors caused by histamines or chloroquine and also enhanced pain-related behaviors caused by capsaicin (Pavlenko et al., 2023). Peripheral injections of S100A8/A9 proteins released by neutrophils prevented the development of anti-inflammatory drug-induced long-term pain (Parisien et al., 2022). In human nucleus pulposus (NP) cells, S100A8/A9 increases the expression of MMP-3/13, TNF-ɑ, IL-6, and inhibits the expression of agglutinin and collagen II. In addition, S100a8/a9 inhibitors prevent disc degeneration (IVDD) and inflammation-related pain in rats (Blom et al., 2020; Zheng et al., 2022).

The Postn gene encodes a secreted extracellular matrix protein that plays a role in tissue development and regeneration, including wound healing and ventricular remodeling after myocardial infarction (Qiao et al., 2024). SELP is an adhesion molecule that belongs to the selectin protein family and is expressed by different cell types such as platelets, endothelial cells, and immune cells. The high expression of SELP in activated platelets makes it an important part of the pathogenesis of thrombosis, particularly in cancer-associated thrombosis (CAT). Furthermore, it has been demonstrated that SELP contributes to tumor–host interactions and cancer immunity (Yeini and Satchi-Fainaro, 2022).

Through differential gene expression analysis and hub gene screening, we identified *Il1β*, *Ptprc*, *Cd4*, *Tlr4*, *Ccl2*, *Tlr2*, *Cxcl10*, *Stat1*, *Cd68*, and *Mmp9* as key hub genes that significantly upregulated and may contribute to the development of chronic orofacial inflammatory pain. Moreover, *Ccl2* is also present in the overlapping DEG genes. It is interesting to note that in the somatic inflammatory pain model, the hub genes *Ccl5*, *Cxcr2*, *Lcn2*, *Spp1*, *Ccr1*, *Mmp8*, *Selp*, and *Cd19* were significantly upregulated, while *Ccl2* and *Fos* were significantly downregulated. Furthermore, five genes *Lcn2*, *Spp1*, *Ccr1*, *Ccl2,* and *Selp* are present in both the DEGs and the hub genes of the somatic inflammatory pain model.

Inflammatory pain is a multifaceted clinical process involving the interaction between the neurological and the immune systems, frequently linked to tissue injury. Cytokines such as IL-1β and TNF play a critical role in the initiation and maintenance of inflammatory pain and are involved in pain signaling at the peripheral and central levels through multiple mechanisms (Chu et al., 2020). Current inflammatory pain research also includes the discussion of neuroplasticity, which involves sensitization of nociceptors, changes in central nervous pathways, and substantive changes in functional alterations. These changes lead to normal innocuous stimuli causing pain, known as central sensitization (Tanaka et al., 2021). However, the mechanism of the persistence and chronicity of inflammatory pain is not fully understood.

The thalamic transcriptome in chronic orofacial versus somatic inflammatory pain reveals a shared neuroinflammatory signature—driven by glia and immune mediators like *S100a8*—that underlies central sensitization, a hallmark of chronic pain. Notably, the chemokine *Ccl2* exhibits divergent thalamic expression between pain types, positioning it as a key candidate for pain-origin-specific circuit modulation, aligning with emerging views of chemokine-driven neuron-microglial crosstalk in pain chronification. While both pain states engage common innate immune and danger-signaling pathways (e.g., *Zbp1* and *Lcn2*), they recruit distinct hub gene networks, suggesting the involvement of different thalamic immune subpopulations and neuro-immune interfaces. These findings underscore that chronic inflammatory pain is not a monolithic neuroimmune state but is shaped by the anatomical origin of nociceptive input, leading to partially specialized thalamic inflammatory landscapes. Future therapeutic strategies could therefore target both the shared neuroinflammatory axis for broad analgesia and origin-specific signals like CCL2/CCR2 for precision pain management.

RT-qPCR validation confirmed that the expression levels of *Ccr1*, *Cxcl13*, *Ccl2*, *Lcn2,* and *S100a8* were all significantly upregulated in the thalamus of the orofacial inflammatory pain model. Similarly, in the somatic inflammatory pain model, the expression of *Ccr1*, *Cxcl13*, *Lcn2*, and *S100a8* was elevated, whereas Ccl2 was uniquely downregulated, perfectly aligning with the bioinformatic screening data. Furthermore, Western blot analysis demonstrated that the protein levels of CCR1 and CXCL13 were significantly increased in CFA-injected rats compared with controls, confirming that these transcriptional alterations successfully translate into functional protein expression. Collectively, these findings reinforce the biological relevance of the identified hub genes and support their potential roles in thalamic neuroimmune modulation during chronic inflammatory pain.

When interpreting the findings of the present research, several limitations should be taken into consideration. First, the integrated datasets originate from different species, different pain sites, and different model durations. Nevertheless, our comparative aimed at delineating candidate genes consistently altered across the two models, as well as the differences that may suggest specific pain modality-specific circuit regulation. We clearly interpret the overlapping differentially expressed genes as candidate genes for the general inflammatory pain pathway, while the unique differentially expressed genes are interpreted as preliminary clues for specific pain types. Second, our behavioral assessments were restricted to von Frey filaments, which measure evoked mechanical pain threshold and reflect the reflexive response to external stimuli. Thus, our transcriptomic profiles may preferentially align with mechanisms of evoked hypersensitivity rather than persistent or spontaneous pain states. Future investigations should incorporate non-evoked pain assays (such as conditioned place preference or grimace scales) to systematically characterize the thalamic substrates of ongoing pain. Third, although we validated protein levels for selected targets (CCR1 and CXCL13), the complete set of hub genes still requires comprehensive proteomic and spatial mapping. Additionally, our Western blot validation was performed with a relatively small sample size; these statistically significant results should be interpreted as preliminary and require validation in larger cohorts. Fourth, our analysis is correlational and does not establish causal relationships between these genes and pain behaviors. Functional intervention experiments, such as localized drug antagonism, antisense oligonucleotides, or CRISPR-mediated knockdown, should be conducted to test whether they directly cause hyperalgesia in orofacial and somatic inflammatory pain. Finally, our transcriptomic analysis was conducted using whole thalamus tissue. Although this global lens captures cross-modal interactions and widespread plasticity, future studies utilizing precise anatomical microdissection are necessary to delineate sub-nucleus-specific molecular dynamics.

## 5 Conclusion

In summary, based on the differential expression analysis of the thalamic transcriptome and the protein level validation, this study discovered the common neuroinflammatory transcriptional characteristics of chronic orofacial and somatic inflammatory pain, with the core manifestation being the coordinated activation of immune and glial-cell-related genes. In the transcriptome screening, multiple overlapping differentially expressed genes were identified after FDR correction (such as *Lcn2*, *Cxcl13*, etc.). Among them, the protein expression of CXCL13 and its receptor CCR1 was significantly upregulated in the CFA-induced inflammatory pain model, which was consistent with the transcriptional direction, confirming that they are reliable molecular markers for thalamic sensitization. Furthermore, although some of the biological candidate genes (such as *S100a8*) did not pass the strict multiple correction, their consistent upward trend and high connectivity in the protein interaction network suggest that they may still be involved in pain regulation. Notably, *Ccl2* exhibited divergent expression between the two pain models, suggesting a potential pain-origin-specific transcriptional signature that warrants separate functional investigation. These findings support the existence of shared thalamic immune-related changes across pain types, while also revealing pain-type-specific molecular signals. Future studies with matched species and time points, combined with targeted functional interventions (e.g., gene knockdown or pharmacological blockade), are needed to establish causal relationships and to inform precision pain management strategies that address both common neuroinflammatory pathways and origin-specific chemokine signaling.

## Data Availability

The original contributions presented in the study are included in the article/Supplementary Material, further inquiries can be directed to the corresponding author.

## Footnotes

1
https://www.ncbi.nlm.nih.gov/geo/

2
http://www.ehbio.com/test/venn/#/

3
https://david.ncifcrf.gov/

4
https://string-db.org

5
https://www.cytoscape.org

## References

[ref1] Ahmadian-Moghadam H. Sadat-Shirazi M. S. Zarrindast M. R. (2018). Cocaine- and amphetamine-regulated transcript (CART): a multifaceted neuropeptide. Peptides 110, 56–77. doi: 10.1016/j.peptides.2018.10.008, 30391426

[ref2] An N. Zhang Y. Xie J. Li J. Lin J. Li Q. . (2024). Study on the involvement of microglial S100A8 in neuroinflammation and microglia activation during migraine attacks. Mol. Cell. Neurosci. 130:103957. doi: 10.1016/j.mcn.2024.103957, 39111720

[ref3] Blom A. B. van den Bosch M. H. Blaney Davidson E. N. Roth J. Vogl T. van de Loo F. A. . (2020). The alarmins S100A8 and S100A9 mediate acute pain in experimental synovitis. Arthritis Res. Ther. 22:199. doi: 10.1186/s13075-020-02295-9, 32854769 PMC7457270

[ref4] Bonin E. A. C. Lejeune N. Szymkowicz E. Bonhomme V. Martial C. Gosseries O. . (2023). Assessment and management of pain/nociception in patients with disorders of consciousness or locked-in syndrome: a narrative review. Front. Syst. Neurosci. 17:1112206. doi: 10.3389/fnsys.2023.1112206, 37021037 PMC10067681

[ref5] Chamaa F. Chebaro M. Safieh-Garabedian B. Saadeh R. Jabbur S. J. Saadé N. E. (2016). Transcriptional expression of inflammatory mediators in various somatosensory relay centers in the brain of rat models of peripheral mononeuropathy and local inflammation. J. Neuroimmunol. 297, 81–91. doi: 10.1016/j.jneuroim.2016.05.005, 27397080

[ref6] Chen H. Tao L. Liang J. Pan C. Wei H. (2023). Ubiquitin D promotes the progression of rheumatoid arthritis via activation of the p38 MAPK pathway. Mol. Med. Rep. 27:53. doi: 10.3892/mmr.2023.12940, 36660934 PMC9879075

[ref7] Chen L. P. Gui X. D. Tian W. D. Kan H. M. Huang J. Z. Ji F. H. (2024). Botulinum toxin type A-targeted SPP1 contributes to neuropathic pain by the activation of microglia pyroptosis. World J Psychiatry 14, 1254–1266. doi: 10.5498/wjp.v14.i8.1254, 39165552 PMC11331382

[ref8] Chu L. W. Cheng K. I. Chen J. Y. Cheng Y. C. Chang Y. C. Yeh J. L. . (2020). Loganin prevents chronic constriction injury-provoked neuropathic pain by reducing TNF-α/IL-1β-mediated NF-κB activation and Schwann cell demyelination. Phytomedicine 67:153166. doi: 10.1016/j.phymed.2019.153166, 31955133

[ref9] Cui M. Ji R. Song L. Wang X. Pan X. Han Y. . (2024). Neuronal and molecular mechanisms underlying chronic pain and depression comorbidity in the paraventricular thalamus. J. Neurosci. 44:e1752232024. doi: 10.1523/jneurosci.1752-23.2024, 38378273 PMC10977023

[ref10] Fruhstorfer H. Gross W. Selbmann O. (2001). von Frey hairs: new materials for a new design. Eur. J. Pain 5, 341–342. doi: 10.1053/eujp.2001.0250, 11558991

[ref11] Furuichi K. Wada T. Sakai N. Iwata Y. Yoshimoto K. Shimizu M. . (2000). Distinct expression of CCR1 and CCR5 in glomerular and interstitial lesions of human glomerular diseases. Am. J. Nephrol. 20, 291–299. doi: 10.1159/000013603, 10970982

[ref12] Galdino G. Veras F. P. Dos Anjos-Garcia T. (2024). The role of the thalamus in nociception: important but forgotten. Brain Sci. 14:741. doi: 10.3390/brainsci14080741, 39199436 PMC11352386

[ref13] Grace P. M. Tawfik V. L. Svensson C. I. Burton M. D. Loggia M. L. Hutchinson M. R. (2021). The Neuroimmunology of chronic pain: from rodents to humans. J. Neurosci. 41, 855–865. doi: 10.1523/jneurosci.1650-20.2020, 33239404 PMC7880288

[ref14] Hong J. Li J. N. Wu F. L. Bao S. Y. Sun H. X. Zhu K. H. . (2023). Projections from anteromedial thalamus nucleus to the midcingulate cortex mediate pain and anxiety-like behaviors in mice. Neurochem. Int. 171:105640. doi: 10.1016/j.neuint.2023.105640, 37951541

[ref15] Joshi R. Brezani V. Mey G. M. Guixé-Muntet S. Ortega-Ribera M. Zhuang Y. . (2024). IRF3 regulates neuroinflammatory responses and the expression of genes associated with Alzheimer's disease. J. Neuroinflammation 21:212. doi: 10.1186/s12974-024-03203-7, 39215356 PMC11363437

[ref16] Kwiatkowski K. Piotrowska A. Rojewska E. Makuch W. Mika J. (2017). The RS504393 influences the level of nociceptive factors and enhances opioid analgesic potency in neuropathic rats. J. Neuroimmune Pharmacol. 12, 402–419. doi: 10.1007/s11481-017-9729-6, 28337574 PMC5527054

[ref17] Lei J. Tang L. L. Jing R. You H. J. (2024). Antinociceptive role of the thalamic dopamine D3 receptor in descending modulation of intramuscular formalin-induced muscle nociception in a rat model of Parkinson's disease. Exp. Neurol. 379:114846. doi: 10.1016/j.expneurol.2024.114846, 38879111

[ref18] Li D. Li Y. C. Zhu Z. Y. Zhang F. C. Zhao Q. Y. Jiang J. H. . (2025). The paraventricular thalamus mediates visceral pain and anxiety-like behaviors via two distinct pathways. Neuron 113, 2310–2324.e7. doi: 10.1016/j.neuron.2025.04.019, 40345185

[ref19] Li Z. Zhang L. Zhang F. Yue L. Hu L. (2024). Deciphering authentic nociceptive thalamic responses in rats. Research 7:0348. doi: 10.34133/research.0348, 38617991 PMC11014087

[ref20] Liang H. Y. Chen Z. J. Xiao H. Lin Y. H. Hu Y. Y. Chang L. . (2020). nNOS-expressing neurons in the vmPFC transform pPVT-derived chronic pain signals into anxiety behaviors. Nat. Commun. 11:2501. doi: 10.1038/s41467-020-16198-5, 32427844 PMC7237711

[ref21] Ma L. Yu L. Jiang B. C. Wang J. Guo X. Huang Y. . (2021). ZNF382 controls mouse neuropathic pain via silencer-based epigenetic inhibition of Cxcl13 in DRG neurons. J. Exp. Med. 218:e20210920. doi: 10.1084/jem.20210920, 34762123 PMC8590274

[ref22] Ma Y. Qin G. H. Guo X. Hao N. Shi Y. Li H. F. . (2022). Activation of δ-opioid receptors in anterior cingulate cortex alleviates affective pain in rats. Neuroscience 494, 152–166. doi: 10.1016/j.neuroscience.2022.05.008, 35569643

[ref23] McMahon S. B. La Russa F. Bennett D. L. (2015). Crosstalk between the nociceptive and immune systems in host defence and disease. Nat. Rev. Neurosci. 16, 389–402. doi: 10.1038/nrn3946, 26087680

[ref24] Mendell L. M. (2014). Constructing and deconstructing the gate theory of pain. Pain 155, 210–216. doi: 10.1016/j.pain.2013.12.010, 24334188 PMC4009371

[ref25] Miyazaki Y. Vieira-de-Abreu A. Harris E. S. Shah A. M. Weyrich A. S. Castro-Faria-Neto H. C. . (2014). Integrin αDβ2 (CD11d/CD18) is expressed by human circulating and tissue myeloid leukocytes and mediates inflammatory signaling. PLoS One 9:e112770. doi: 10.1371/journal.pone.0112770, 25415295 PMC4240710

[ref26] Ou Y. Ni X. Gao X. Yu Y. Zhang Y. Wang Y. . (2024). Structural and functional changes of anterior cingulate cortex subregions in migraine without aura: relationships with pain sensation and pain emotion. Cereb. Cortex 34:bhae040. doi: 10.1093/cercor/bhae040, 38342690 PMC10859245

[ref27] Parisien M. Lima L. V. Dagostino C. El-Hachem N. Drury G. L. Grant A. V. . (2022). Acute inflammatory response via neutrophil activation protects against the development of chronic pain. Sci. Transl. Med. 14:eabj9954. doi: 10.1126/scitranslmed.abj9954, 35544595 PMC10317000

[ref28] Pavlenko D. Todurga Seven Z. Bystrom L. Markan A. Verpile R. Ishida H. . (2023). Crisaborole inhibits itch and pain by preventing neutrophil infiltration in a mouse model of atopic dermatitis. Acta Derm. Venereol. 103:adv13382. doi: 10.2340/actadv.v103.13382, 37605895 PMC10461178

[ref29] Pawlik K. Ciapała K. Ciechanowska A. Kwiatkowski K. Mika J. (2022). Pharmacological evidence of the important roles of CCR1 and CCR3 and their endogenous ligands CCL2/7/8 in hypersensitivity based on a murine model of neuropathic pain. Cells 12:98. doi: 10.3390/cells12010098, 36611891 PMC9818689

[ref30] Pinho-Ribeiro F. A. Verri W. A. Jr. Chiu I. M. (2017). Nociceptor sensory neuron-immune interactions in pain and inflammation. Trends Immunol. 38, 5–19. doi: 10.1016/j.it.2016.10.001, 27793571 PMC5205568

[ref31] Qiao B. Liu X. Wang B. Wei S. (2024). The role of periostin in cardiac fibrosis. Heart Fail. Rev. 29, 191–206. doi: 10.1007/s10741-023-10361-y, 37870704

[ref32] Qu Y. Cai R. Li Q. Wang H. Lu L. (2024). Neuroinflammation signatures in dorsal root ganglia following chronic constriction injury. Heliyon 10:e31481. doi: 10.1016/j.heliyon.2024.e31481, 38813203 PMC11133895

[ref33] Robertson R. V. Meylakh N. Crawford L. S. Tinoco Mendoza F. A. Macey P. M. Macefield V. G. . (2024). Differential activation of lateral parabrachial nuclei and their limbic projections during head compared with body pain: a 7-tesla functional magnetic resonance imaging study. NeuroImage 299:120832. doi: 10.1016/j.neuroimage.2024.120832, 39236852

[ref34] Rotpenpian N. Yakkaphan P. (2021). Review of literatures: physiology of orofacial pain in dentistry. eNeuro 8:ENEURO.0535-20.2021. doi: 10.1523/eneuro.0535-20.2021, 33820801 PMC8086974

[ref35] Tanaka M. Török N. Tóth F. Szabó Á. Vécsei L. (2021). Co-players in chronic pain: Neuroinflammation and the tryptophan-kynurenine metabolic pathway. Biomedicine 9:897. doi: 10.3390/biomedicines9080897, 34440101 PMC8389666

[ref36] Tu Y. Li Z. Zhang L. Zhang H. Bi Y. Yue L. . (2024). Pain-preferential thalamocortical neural dynamics across species. Nat. Hum. Behav. 8, 149–163. doi: 10.1038/s41562-023-01714-6, 37813996

[ref37] Vega-Ramírez M. T. Becerril-Villanueva E. Maldonado-García J. L. Pavón L. Pérez-Sánchez G. (2024). S100 proteins: a new frontier in fibromyalgia research. Mol. Brain 17:29. doi: 10.1186/s13041-024-01102-9, 38797848 PMC11129469

[ref38] Wang B. Wang L. N. Wu B. Guo R. Zhang L. Zhang J. T. . (2024). Astrocyte PERK and IRE1 signaling contributes to morphine tolerance and hyperalgesia through upregulation of Lipocalin-2 and NLRP3 Inflammasome in the rodent spinal cord. Anesthesiology 140, 558–577. doi: 10.1097/aln.0000000000004858, 38079113

[ref39] Wang J. Yin C. Pan Y. Yang Y. Li W. Ni H. . (2023). CXCL13 contributes to chronic pain of a mouse model of CRPS-I via CXCR5-mediated NF-κB activation and pro-inflammatory cytokine production in spinal cord dorsal horn. J. Neuroinflammation 20:109. doi: 10.1186/s12974-023-02778-x, 37158939 PMC10165831

[ref40] Wu W. Zhou Q. Masubuchi T. Shi X. Li H. Xu X. . (2020). Multiple signaling roles of CD3ε and its application in CAR-T cell therapy. Cell 182, 855–871.e23. doi: 10.1016/j.cell.2020.07.018, 32730808

[ref41] Xu M. Wang Y. Xia R. Wei Y. Wei X. (2021). Role of the CCL2-CCR2 signalling axis in cancer: mechanisms and therapeutic targeting. Cell Prolif. 54:e13115. doi: 10.1111/cpr.13115, 34464477 PMC8488570

[ref42] Yeini E. Satchi-Fainaro R. (2022). The role of P-selectin in cancer-associated thrombosis and beyond. Thromb. Res. 213, S22–S28. doi: 10.1016/j.thromres.2021.12.027, 36210556

[ref43] Yosten G. L. Harada C. M. Haddock C. Giancotti L. A. Kolar G. R. Patel R. . (2020). GPR160 de-orphanization reveals critical roles in neuropathic pain in rodents. J. Clin. Invest. 130, 2587–2592. doi: 10.1172/jci133270, 31999650 PMC7190928

[ref44] Zhang C. Chen R. X. Zhang Y. Wang J. Liu F. Y. Cai J. . (2017). Reduced GABAergic transmission in the ventrobasal thalamus contributes to thermal hyperalgesia in chronic inflammatory pain. Sci. Rep. 7:41439. doi: 10.1038/srep41439, 28150719 PMC5288727

[ref45] Zhang Y. Wang Y. Zhao W. Li L. Li L. Sun Y. . (2022). Role of spinal RIP3 in inflammatory pain and electroacupuncture-mediated analgesic effect in mice. Life Sci. 306:120839. doi: 10.1016/j.lfs.2022.120839, 35902029

[ref46] Zheng J. Wang J. Liu H. Chen F. Wang H. Chen S. . (2022). Alarmins S100A8/A9 promote intervertebral disc degeneration and inflammation-related pain in a rat model through toll-like receptor-4 and activation of the NF-κB signaling pathway. Osteoarthr. Cartil. 30, 998–1011. doi: 10.1016/j.joca.2022.03.011, 35405347

